# Bortezomib in combination with celecoxib in patients with advanced solid tumors: a phase I trial

**DOI:** 10.1186/1471-2407-7-221

**Published:** 2007-12-03

**Authors:** John Hayslip, Uzair Chaudhary, Mark Green, Mario Meyer, Steven Dunder, Carol Sherman, Shanta Salzer, Andrew Kraft, Alberto J Montero

**Affiliations:** 1 Hollings Cancer Center, Medical University of South Carolina, Charleston, South Carolina, USA

## Abstract

**Background:**

COX-2 inhibitors, such as celecoxib, and ubiquitin-proteasome pathway inhibitors, such as bortezomib, can down-regulate NF-κB, a transcription factor implicated in tumor growth. The objective of this study was to determine the maximum tolerated dose and dose-limiting toxicities of bortezomib in combination with celecoxib in patients with advanced solid tumors.

**Methods:**

Patients received escalating doses of bortezomib either on a weekly schedule (days 1, 8, 15, 22, and 29 repeated every 42 days) or on a twice-weekly administration schedule (days 1, 4, 8, and 11 repeated every 21 days), in combination with escalating doses of celecoxib twice daily throughout the study period from 200 mg to 400 mg twice daily.

**Results:**

No dose-limiting toxicity was observed during the study period. Two patients had stable disease lasting for four and five months each, and sixteen patients developed progressive disease.

**Conclusion:**

The combination of bortezomib and celecoxib was well tolerated, without dose limiting toxicities observed throughout the dosing ranges tested, and will be studied further at the highest dose levels investigated.

**Trial registration number:**

NCT00290680.

## Background

The ubiquitin-proteasome pathway plays an important role in cell cycle regulation, neoplastic growth, and metastasis [1,2]. At the heart of this degradative pathway is the 26S proteasome, an adenosine triphosphate-dependent protease. The 26S proteasome consists of a core 20S particle, which contains the catalytic proteinase functions, symmetrically bound to two copies of the regulatory 19S particle. Proteolytic removal of damaged or misfolded ubiquitinated proteins is an important part of the homeostatic function of the 26S proteasome. Additionally, the 26S proteasome is vital in degrading regulatory proteins that govern cell cycle, transcription factor activation, cell trafficking, and apoptosis.

The ubiquitin-proteasome pathway is responsible for the ordered degradation of several regulatory proteins necessary for cells to progress through the cell cycle. The tumor suppressor p53, which acts as a negative regulator of cell growth, is one example of targeted ubiquitin-proteasome mediated degradation. p53 is required for the transcription of a number of genes involved in cell cycle control and DNA synthesis. p53 also plays an important role in apoptosis induced by cellular damage [3]. Cyclins and the cyclin-dependent kinase inhibitors p21 and p27 are also regulated by proteasome-dependent proteolysis [4]. Both p21 and p27 can induce cell cycle arrest by inhibiting the cyclin D-, E-, and A-dependent kinases [5].

The ubiquitin-proteasome pathway also plays an important role in transcriptional regulation. Nuclear factor-κB (NF-κB) is a key transcription factor, whose activation is regulated by proteasome-mediated degradation of the inhibitor protein I kappa B alpha-associated protein kinase (IκBa) [4,5]. Cell adhesion molecules such as E-selectin, ICAM-1, and VCAM-1, are regulated by NF-κB and are involved in the development of angiogenesis and tumor metastasis in vivo [4]. During metastasis, these molecules direct the adhesion and extravasation of tumor cells from the vasculature to distant tissue sites within the body. Additionally, many cell types require NF-κB to maintain cell viability as an anti-apoptotic controlling factor or growth factor [4]. Inhibiting NF-κB activation by stabilizing the IκBa protein potentiates apoptosis of cancer cells to environmental stresses and cytotoxic agents.

Another enzyme recently targeted for cancer prevention and treatment, cyclooxygenase (COX)-2, has garnered increasing interest due to substantial epidemiologic, experimental, pathologic, and clinical evidence suggesting that nonsteroidal anti-inflammatory drugs (NSAIDs) possess anticancer properties [6-8]. NSAIDs are thought to exert their anti-inflammatory effects by inhibiting prostaglandin synthesis through nonspecific inhibition of COX enzymes. Prostaglandins, in particular prostaglandin E2, appear to be important in oncogenesis due to their effects on cellular adhesion, immune surveillance, and apoptosis [9]. Compared with normal tissues, cancers have been shown to over-express prostaglandins [10-17]. In several animal and human models, inhibiting prostaglandin synthesis by blocking COX-2 appears to protect against oncogenesis in many tissue types including: breast, colon, esophageal, lung, skin, and head and neck cancers [6,7,10-16].

COX-2 is undetectable in most normal tissues; however, it is induced at sites of inflammation by cytokines, growth factors, tumor promoters, and is over-expressed in neoplasia [17]. Several mechanisms have been proposed to explain the role of COX-2 in tumorigenesis, including stimulating cancer cell proliferation, enhancing angiogenesis, and inhibiting apoptosis [18,19]. In vitro inhibition of COX-2 with targeted molecules is thought to promote apoptosis of cancer cells through inhibition of NF-kB activation [20].

Both COX-2 inhibitors and ubiquitin-proteasome pathway inhibitors down-regulate NF-κB and their combination is an interesting opportunity to explore clinical synergy with these two classes of agents. The ubiquitin-proteasome pathway is involved in NF-κB regulation through its impact on degradation of IκB and release of NF-κB for translocation from the cytoplasm to the nucleus [21]. Inhibition of IκB degradation by proteasome inhibition can limit the actions of NF-κB rendering tumor cells more prone to apoptosis. The combination of the NSAID sulindac and bortezomib was found to be synergistic both *in vitro*, in colon cancer cell lines, and *in vivo*, in a xenograft model [22]. Additionally, the COX-2 inhibitor celecoxib has been reported to induce apoptosis independent of its COX-2 effects using NF-κB as a probably target [23]. Consequently, given the relevance of the COX-2 oncogene to numerous solid tumors, clinical evaluation of a COX-2 inhibitor/proteasome inhibitor combination is warranted in patients with solid tumors.

We therefore hypothesized that the combination of the COX-2 inhibitor celecoxib and the ubiquitin-proteasome pathway inhibitor bortezomib is a potentially active clinical combination in the treatment of advanced solid tumors. Therefore, we conducted a phase I study to determine the maximum tolerated dose (MTD) and toxicity profile of bortezomib in combination with celecoxib.

## Methods

### Patient Selection

Patients with a stage IV histologically proven malignant neoplasm (solid tumor) arising from any primary site with the exception of bone marrow or lymphoid tissue were eligible for this study. Other eligibility criteria included: recurrent or progressive disease after chemotherapy or radiation or chemotherapy or radiotherapy-naive disease that, due to the patient co-morbidities or wishes, is not a candidate for standard treatment, no prior therapy with bortezomib, Eastern Cooperative Oncology Group (ECOG) performance status of 2 or less, neutrophil count > 1500/mm3, platelets > 100,000/mm3, creatinine < 2.0 mg/dl (or creatinine clearance of > 30 ml/minute), bilirubin < 2 mg/dl, and age > 18 years. Previous radiotherapy was permitted provided that it was completed more than two weeks before enrollment. All subjects provided written informed consent.

The exclusion criteria were as follows: ≥ grade 2 peripheral neuropathy, hypersensitivity to bortezomib, boron, or mannitol, hypersensitivity to any of the COX-2 inhibitors, hypersensitivity to sulfa drugs, hypersensitivity to other NSAIDs, active gastrointestinal ulcer, or history of GI bleeding resulting from prior therapy with NSAIDs, pregnancy, or actively breast-feeding. This study was approved by the institutional review board of the Medical University of South Carolina.

### Study Design

This was an open-label, single center, nonrandomized, dose-escalating phase I study utilizing a modified Fibonacci design. Escalating doses of bortezomib were administered as a short IV bolus on days 1, 4, 8, and 11 every 3 weeks or on days 1, 8, 15, 22 and 29 every 6 weeks along with celecoxib orally twice a day. Each cycle was 21 days or 42 days in length depending on the bortezomib administration assignment. The trial did not escalate above 1.3 mg/m^2 ^of bortezomib twice weekly or 1.6 mg/m^2 ^of bortezomib weekly because previously reported single-agent bortezomib trials have reported significant toxicities above these doses [24,25]. Similarly, celecoxib was not escalated above 400 mg twice daily due to emerging cardiac toxicity data from other trials [26]. Three patients were initially enrolled at each dosing level. If one patient experienced a dose-limiting toxicity, an additional three patients would be accrued to that level. However, if two patients experience a dose-limiting toxicity no further patients would be accrued to that dose level. No maximal number of cycles was pre-specified and patients with stable or responding disease were encouraged to remain on therapy in the absence of significant toxicites. For patients who experienced a dose limiting toxicity (DLT), a dose adjustment was allowed if it was felt that the patient was benefiting from the therapy (responding or stable disease). The dose of each drug in this study is detailed in Table 1.

**Table 1:** Bortezomib and celecoxib cohort dosing schedules

Cohorts	Bortezomib (mg/m^2^)	Bortezomib days of administration	Celecoxib (mg PO bid)
Level 1	1.0	1, 8, 15, 22, 29 q42 days	200
Level 2	1.0	1, 4, 8, 11 q21 days	200
Level 3	1.3	1, 8, 15, 22, 29 q42 days	300
Level 4	1.3	1, 4, 8, 11 q21 days	300
Level 5	1.6	1, 8, 15, 22, 29 q42 days	400
Level 6	1.3	1, 4, 8, 11 q21 days	400

### Definition of dose-limiting toxicities and maximum tolerated dose

Only dose-limiting toxicities (DLTs) occurring during the first cycle of therapy were used to define the maximum tolerated dose (MTD). DLTs were defined as follows: any documented grade IV granulocytopenia or thrombocytopenia possibly or probably due to protocol therapy, any documented ≥ grade III non-hematologic toxicity (except for grade III nausea or vomiting or diarrhea in the first cycle but controlled to a maximum of grade II with anti-emetic or anti-diarrheal therapy in a second cycle), inability to deliver any chemotherapy on day 8 of cycle 1 because of neutrophil count < 1000/μl or platelet count < 50,000/μl, or the inability to begin a subsequent cycle of chemotherapy within 14 days of the scheduled date due to persisting hematologic or non-hematologic toxicity of greater than grade 1.

### Treatment Assessments

Response and progression were evaluated in this study using the international guidelines proposed by the Response Evaluation Criteria in Solid Tumors (RECIST) Committee [27]. Baseline evaluations included physical exam, serum chemistries, hepatic function testing, complete blood counts, and computed tomography or magnetic resonance imaging at the discretion of the treating physician. The same imagining modality and laboratory evaluations were completed every two cycles to evaluate for response or progression. Patients underwent a physical exam, serum chemistries, and complete blood count evaluation on the first day of each cycle. Additionally, a complete blood count was required on day 4 of the 21 day cycles or day 8 of the 42 days cycles. Further, complete blood count and serum chemistries were required on day 8 of the 21 day cycles or day 15 of the 42 day cycles. NCI Common Toxicity Criteria Adverse Event version 3.0 criteria were used to grade toxicities.

## Results

### Patient characteristics

Eighteen patients were enrolled from April 2005 to July 2006. All patients met the entry criteria and were treated in accordance with protocol guidelines. The median age of patients was 62 years and both males and females were well represented. All patients had performance status of 2 or better and patients with a variety of tumor histologies were enrolled (Table 2).

**Table 2:** Patient characteristics

Patients enrolled	18
Sex	
Male	10
Female	8
Age, years	
Median	62
Range	46–77
ECOG status	
0	4
1	12
2	2
Cancer Type	
Adrenocortical	1
Colon	2
Esophageal	1
Fallopian Tube	1
Hepatocellular	1
Leiomyosarcoma	1
Non-Small Cell Lung Carcinoma	1
Ovarian	1
Pancreatic	3
Renal Cell	2
Squamous Cell	2
Transitional Cell	1
Unknown Primary	1
Previous lines of chemotherapy	
0–3	11
4–6	5
>6	2

### Toxicity and treatment cycles

No dose limiting toxicities were observed during the first cycle of treatment for any patient and the maximal planned dosages were achieved. Creatinine elevation, neuropathy, and fatigue/weakness were the most commonly observed toxicities and a full toxicity report is included (Table 3). From these results, we did not discern specific synergistic toxicities from this combination and dose level six has been selected for the subsequent phase II study. The median number of cycles was two for cohort one, two for cohort two, one for cohort three, two for cohort four, one for cohort five, and two for cohort six. One patient experienced stable disease for six cycles until experiencing diarrhea, thought to be bacterial-induced, and withdrew from the study. All other patients ended treatment due to disease progression and no other patients withdrew or were removed due to toxicities.

**Table 3:** Incidence of clinically relevant toxicities

Toxicities	Cohort 1		Cohort 2		Cohort 3		Cohort 4		Cohort 5		Cohort 6	
	Toxicity Grade											
	1/2	3/4	1/2	3/4	1/2	3/4	1/2	3/4	1/2	3/4	1/2	3/4

Alkaline phosphatase elevation	0	0	0	0	0	0	0	1	0	0	0	0
Anemia	0	0	1	0	0	0	0	0	0	0	0	0
Creatinine elevation	1	0	2	0	0	0	0	0	0	0	0	0
Edema	0	0	0	0	0	0	0	0	0	0	1	0
Hyperkalemia	1	0	0	0	0	0	0	0	0	0	0	0
Hypoalbuminemia	0	0	1	0	0	0	0	0	0	0	0	0
Infection	0	0	0	1*	0	0	0	0	0	0	0	0
Insomnia	0	0	0	0	1	0	0	0	0	0	0	0
Nausea/emesis	0	0	0	0	1	0	0	0	0	0	0	1
Neuropathy	0	0	1	0	0	0	1	0	0	0	0	0
Pruritis	0	0	1	0	0	0	0	0	0	0	0	0
Weakness/fatigue	0	0	1	1	0	0	0	0	0	0	0	0

* Right gluteal and inguinal furuncles (culture positive for mixed flora – gram positive and gram cocci as well as methicillin sensitive and resistant staph aureus.

### Tumor response

In this study all patients were evaluable for response by RECIST criteria. Of the eighteen patients studied, sixteen patients experienced progressive disease during the first two cycles. One patient with adrenocortical cancer (cohort 4) and another with renal cell cancer (cohort 2) experienced 4.3 and 5.1 months of stable disease while on protocol therapy.

## Discussion

Both COX-2 inhibitors and ubiquitin-proteasome pathway inhibitors down-regulate NF-κB. Combining these two classes of agents is an interesting opportunity to investigate potential clinical synergy with a combination of drugs not known to have overlapping toxicities.

This is the first phase I study to combine bortezomib and celecoxib in patients with advanced solid tumors. The primary endpoint of this study was to determine the maximal tolerated doses of these agents when taken in combination. No dose-limiting toxicities were observed during the first cycle of treatment at any dose-level and cumulative toxicities were manageable.

In our phase I study, two patients with previously progressive disease experienced stable disease on therapy. A patient with metastatic adrenocortical cancer, previously intolerant of mitotane, achieved stable disease for 4.3 months until progression. A second patient with papillary renal cell carcinoma achieved stable disease for 5.1 months. This patient had documented tumor progression on multi-agent chemotherapy prior to enrolling on this trial.

Interesting new data have been reported for celecoxib and bortezomib since our study began. Celecoxib has been shown to potentially have single-agent activity in men with PSA recurrence after local prostate cancer therapy [28]. Also, patients with non-small cell lung cancer receiving single-agent bortezomib recently reported an 8% response rate [29]. Similarly, a phase II trial of patients with metastatic renal cell carcinoma reported a response rate of 11% and 38% of patients reported stable disease with bortezomib [30]. Conversely, no tumor responses were seen in recent studies of bortezomib in metastatic melanoma, breast cancer, or colorectal cancer [31-33].

## Conclusion

In conclusion, the combination of bortezomib and celecoxib was well tolerated across the dose levels investigated. No dose-limiting toxicities were witnessed and we plan to proceed with dose level six (bortezomib 1.3 mg/m^2 ^days 1, 4, 8, and 11 repeated every 21 days in combination with celecoxib 400 mg orally twice daily) in a subsequent phase II study.

## Competing interests

The pharmaceutical company Millenium provided bortezomib and provided funding for the conduct of this trial. The authors declare that there are no other competing interests.

## Authors' contributions

JH has made substantial contributions to data analysis, interpretation, and article composition and revision; UC has been involved in acquisition of data; MG has been involved in conception and design; MM has been involved in conception and design; SD has been involved with acquisition of data; CS has been involved with acquisition of data; SS has been involved with acquisition of data; AK has been involved in acquisition of data; AJM has made substantial contributions with acquisition of data, analysis and interpretation of data, and article composition and revision. All authors have read and approved the final manuscript.

## Pre-publication history

The pre-publication history for this paper can be accessed here:


